# Large-Scale Genomics Reveals the Genetic Characteristics of Seven Species and Importance of Phylogenetic Distance for Estimating Pan-Genome Size

**DOI:** 10.3389/fmicb.2019.00834

**Published:** 2019-04-24

**Authors:** Sang-Cheol Park, Kihyun Lee, Yeong Ouk Kim, Sungho Won, Jongsik Chun

**Affiliations:** ^1^Institute of Health and Environment, Seoul National University, Seoul, South Korea; ^2^Department of Systems Biotechnology, Chung-Ang University, Anseong, South Korea; ^3^Interdisciplinary Program in Bioinformatics, Seoul National University, Seoul, South Korea; ^4^Department of Public Health Sciences, Seoul National University, Seoul, South Korea; ^5^Department of Biological Sciences and Institute of Molecular Biology and Genetics, Seoul National University, Seoul, South Korea

**Keywords:** pan-genome, core-genome, Heaps’ law, gene pool, large-scale genomics, seven species, estimation model

## Abstract

For more than a decade, pan-genome analysis has been applied as an effective method for explaining the genetic contents variation of prokaryotic species. However, genomic characteristics and detailed structures of gene pools have not been fully clarified, because most studies have used a small number of genomes. Here, we constructed pan-genomes of seven species in order to elucidate variations in the genetic contents of >27,000 genomes belonging to *Streptococcus pneumoniae*, *Staphylococcus aureus* subsp. *aureus*, *Salmonella enterica* subsp. *enterica*, *Escherichia coli* and *Shigella* spp., *Mycobacterium tuberculosis* complex, *Pseudomonas aeruginosa*, and *Acinetobacter baumannii.* This work showed the pan-genomes of all seven species has open property. Additionally, systematic evaluation of the characteristics of their pan-genome revealed that phylogenetic distance provided valuable information for estimating the parameters for pan-genome size among several models including Heaps’ law. Our results provide a better understanding of the species and a solution to minimize sampling biases associated with genome-sequencing preferences for pathogenic strains.

## Introduction

As genome sequencing becomes more accessible and practical, the primary interests of bacterial genomics have shifted from the sequence to genomic variations, because the genome sequence alone is insufficient to effectively explain biological characteristics. From a macro perspective, studies of genetic variations between organisms have attempted to overcome such limitations and broaden the understanding of relationships between genes and biological traits. Specifically, the recent accumulation of genome sequences introduced the concept of a pan-genome, resulting in its wide application in an attempt to account for the genomic diversity present within a given phylogenetic clade (Tettelin et al., 2005). A pan-genome describes the full complement of all genes known to exist in all members of a given clade and comprises both core and accessory genomes. A core genome is defined as a gene set commonly shared by almost all clade members, whereas an accessory genome is defined as a gene set shared within only one or some strains.

Assuming that the given clade is a species, a pan-genome of this species can be classified into two types (opened or closed) according to its characterization of the gene pool. Openness indicates that the gene pool of a species has no upper limit and is observed when new strains consistently present novel genes. Several species, such as *Escherichia coli*, *Streptococcus agalactiae*, *Leuconostoc mesenteroides*, and *Propionibacterium acnes*, reportedly harbor open pan-genomes (Tettelin et al., 2005; Tomida et al., 2013; Salipante et al., 2015; Chun et al., 2017). On the other hand, closedness indicates that the gene pool of the pan-genome is limited, and that even the appearance of a new strain does not result in growth of the gene pool. Pathogenic and symbiotic species tend to have closed pan-genomes, with *Bacillus anthracis*, *Listeria monocytogenes*, and *Bifidobacterium longum* identified as having this characteristic (Medini et al., 2005; Deng et al., 2010; O’Callaghan et al., 2015). According to results reported by Tettelin et al. (2008), a finite or infinite pan-genome can be determined by a prediction using Heaps’ law and a power law. Heaps’ law is formulated as n = κ*N*^γ^, where n is the pan-genome size, *N* is the number of genomes used, and κ and γ are the fitting parameters. The exponent γ determines whether the pan-genome has an open property. For γ < 0, the pan-genome is closed, and its size approaches a constant as more genomes are used, whereas for γ > 0, the pan-genome is open, and its size increases as more genomes are included. The open property of a given pan-genome can also be estimated by using a power law model according to the formula Δ*n* = κ*N*^-α^, where Δ*n* is the number of newly added genes, *N* is the number of genomes used, and κ and α are the fitting parameters. For α > 1, the pan-genome is closed, and for α < 1, the pan-genome is open. Heaps’ law and power law are mathematically similar.

Multiple studies have investigated the pan-genome size of several species, including *Acinetobacter baumannii* and *Staphylococcus aureus*. However, many of these studies utilized a relatively small number of strains and the estimates of the parameters (α or γ) had large standard errors. Because of these errors, conclusions regarding the openness and closeness of a species were inconsistent. For example, 249 genomes of *A. baumannii* strains were analyzed using a power law by Chan et al. (2015), with the results showing that the species has a closed pan-genome. Controversially, the pan-genome size of 16 strains of the same species was estimated as continuing to increase in size with each additional strain sequenced (Liu et al., 2014). Similarly, *S. aureus* was predicted to have a closed pan-genome according to a power law model, whereas other studies reported that its pan-genome repertoire generated from 64 strains is likely to increase indefinitely as more strains are analyzed (Tettelin et al., 2008; Bosi et al., 2016). Such inconsistencies might be attributable to an insufficient number of genomes used to explain general species properties and/or sampling bias in genome sequencing, such as those associated with clinically significant pathogens.

In this study, we performed a large-scale comparative genome analysis of seven species: *Streptococcus pneumoniae*, *S. aureus* subsp. *aureus*, *Salmonella enterica* subsp. *enterica*, *E. coli*, *Shigella* spp., members of the *Mycobacterium tuberculosis* complex, *Pseudomonas aeruginosa*, and *A. baumannii*. To minimize the standard errors of parameter estimates, we downloaded a large number of genomes (>27,000, at least 1,100 per species) for the seven species from the NCBI GenBank database and explored the genetic diversity among the seven species along with other species-specific genome characteristics. To explore the structure of the gene pool in various taxa, we chose different genus as possible. According to this pan-genome, we described the capacity of the species to acquire exogenous genes. Furthermore, we found that genome phylogeny, as well as the number of genomes, includes useful information about pan-genome size, and suggests a new statistical model to estimate parameters for pan-genome size. We evaluated the proposed methods using cross-validation, finding that the results described the extensibility of the gene pool of species with better accuracy. These analyses provide insight into intra-species variability and their evolutionary processes in the context of prokaryotic genomes.

## Materials and Methods

### Data Preparation and Quality Control

The sequences of >32,000 whole genomes from seven species, including *S. pneumoniae*, *S. aureus* subsp. *aureus*, *S. enterica* subsp. *enterica*, *E. coli* and *Shigella* spp., members of the *M. tuberculosis* complex, *P. aeruginosa*, and *A. baumannii*, were downloaded from NCBI GenBank^1^. Since the draft genomes also were downloaded, to minimize the effect of low-quality sequencing data, genomes that did not satisfy specific criteria (genome sequences having >500 contigs and <20,000 bp of N50 size) were considered low quality and filtered. We then used CheckM to assess quality based on marker genes for the remaining genomes (Parks et al., 2015). After the genomes were assessed, those with a completeness of >99% and a contamination rate <3% were retained for subsequent analysis. In this analysis, *E. coli* and *Shigella* spp. were considered to be the same species. Additionally, all member species in the *M. tuberculosis* complex (MTC or MTBC), including *M. tuberculosis*, *Mycobacterium microti*, *Mycobacterium africanum*, *Mycobacterium caprae*, and *Mycobacterium bovis*, were treated as one species. All the strains used in this study are listed in Supplementary Tables S1–S7.

### Reaffirmation of Taxonomic Information Using Average Nucleotide Identity (ANI)

To ensure that the genomes of each strain indeed belonged to the assigned species, ANI values were calculated for all strains (Goris et al., 2007). The calculation method was implemented as described previously using BLAST version 2.2.29+. Pairwise ANI values between the strains and their type strains were acquired, and the values were averaged and examined to determine those with ANI values >95%, which generally represents a threshold for species delimitation (Richter and Rosselló-Móra, 2009). An ANI value <95% for a given genome was considered to be a misclassified taxonomy and discarded from the dataset, even if the previously assigned taxonomy represented the species.

### Gene Prediction and Functional Annotation

An automatic annotation process was performed to ensure the consistency of gene coordinates, except for *E. coli* K-12 MG1655 (a manually well-annotated strain). The automatic annotation process comprised two steps. First, tRNA was identified using tRNA Scan-SE version 1.3.1^2^, and rRNAs (5S, 16S, and 23S) and other non-coding RNAs were identified by HMMER version 3.1b1^3^ (Schattner et al., 2005; Finn et al., 2011). Putative protein-coding genes were predicted for all individual sequences using Prodigal version 2.6.2 with default parameters (Hyatt et al., 2010). Second, biological function information for each gene was annotated according to categories assigned using the EggNOG 4.5 database^4^ (Powell et al., 2014).

### Construction of the Pan-Genome

Prior to construction of the pan-genome, the predicted coding regions of the sequences were converted to protein sequences. If the protein sequences comprised <25 amino acids or contained ambiguous amino acids in >10% of the sequence, they were ignored. Because the pan-genome was constructed in order to compare genomic features within close strains belonging to the same species, a large proportion of those expected to be nearly identical. Therefore, clustering was conducted with UCLUST^5^, and relatively high standards of sequence identity (≥90%) and coverage (≥90%) were used to detect genes with highly similar sequences (Edgar, 2010). This process resulted in one representative sequence for each cluster. After the first round of clustering, there still remained fragments needing to be included in the same groups. To produce proper protein clusters, another clustering step was performed. When compared with previously generated clusters, the new clusters were formed according to a high identity value (≥95% identity) used as a parameter without considering sequence coverage. In each cluster, the most frequently occurring and longest sequences were selected as the final representative sequences. For simplicity, the term “gene” was used instead of “cluster of proteins” in the remainder of this article.

### Phylogenetic Analysis

To establish a species-tree reference, outgroups were selected as being in the same genus as that of the objective species. We confirmed that the selected outgroup species were not the same species as the target species according to the ANI value and depending upon whether the value was >95%. After outgroup confirmation, BLAST was performed to extract orthologous sequences from the outgroups that were similar to the representative sequences of the pan-genomes. When the coverage and identity were >75 and >70%, respectively, the extracted sequences from the outgroup genomes were integrated into the original sequence clusters. To build a species phylogenetic tree, we selected genes belonging to the strict core genome (defined as genes commonly shared by >99.9% of strains). The gene sequences were aligned using the codon-based algorithm included in MAFFT version 7.215^6^ with default parameters (Katoh and Standley, 2013). Single nucleotide polymorphisms were extracted from these alignments and concatenated to form a multiple sequence alignment. A maximum-likelihood tree was constructed using FastTree2^7^ under the generalized time-reversible model of evolution (Price et al., 2010).

### Estimation of Openness or Closeness

The parameters for pan-genome size can be estimated as a function of the number of genomes using Heaps’ law, as proposed by Tettelin et al. (2008). The Heaps’ law uses the values associated with the genome and pan-genome size as a predictor and outcome, respectively, whereas the power law model uses the value associated with the genome size and the number of newly added gene clusters. For simplicity, only Heaps’ law were applied to all seven species, and estimated parameters with 100 random samples of genome addition orders were used to determine the openness and closeness of the species.

### A Novel Estimation Model Considering Phylogeny

Phylogeny represents genetic distances among species, and if species with large phylogenetic distances are utilized, the pan-genome sizes are expected to be larger. Heaps’ law and power law do not consider such effects, which were accounted for in the proposed method.

Let n and N be the pan-genome size and the number of genomes, respectively. We randomly selected N_i_ genomes and calculated their pan-genome size, n_i_ and the cumulative sum of the branch lengths from the core genome phylogeny, D_i_ (Supplementary Figure S1). We then applied the following linear models:

$$M1:\;\log\;n_{\text{i}}\;\sim \;{\text{β}}_{0}\;+\;{\text{β}}_{1}\;\log N_{\text{i}}$$

$$M2:\;\log\;n_{\text{i}}\;\sim \;{\text{β}}_{0}\;+\;{\text{β}}_{1}\;\log(D_{\text{i}}\;+\;1)$$

$$M3:\;\log\;n_{\text{i}}\;\sim \;{\text{β}}_{0}\;+\;{\text{β}}_{1}\;\log(D_{\text{i}}\;+\;1)\;+\;{\text{β}}_{2}\;\log\;N_{\text{i}}$$

For M1, a similar relationship between n_i_ and N_i_ in the Heaps’ law was expected, except for the normality of the error. Notably, n_i_ was assumed to follow a log-normal distribution for M1 and a normal distribution for the Heaps’ law. We fit M1, M2, and M3 using randomly selected samples, and their mean was used as the final model. These methods were applied to all seven species, followed by the performance of 10-fold cross-validation and calculation of the root mean square errors (RMSEs) using test data. All genomes were divided into 10 different subgroups with equal numbers of genomes. Genome size prediction function was estimated with nine subgroups of the data set. Prediction accuracy of the candidate model is evaluated by measuring the RMSEs between the pan-genome size estimated by the candidate model and the actual pan-genome size.

To identify the effect of the genomes on the model parameters, we examined the fluctuation of each model parameter upon the addition of each genome. In general, the initial size of the pan-genome varies dramatically depending on the number of genomes included; therefore, we did not consider a case where less than five genomes were used. The mean of 100 randomly sampled datasets was used.

## Results

### General Characteristics of the Data

We downloaded >32,000 genomes, with the number of remaining genomes after filtering at 27,656: 6537 for *S. pneumoniae*; 6282 for *S. aureus* subsp. *aureus*, 4527 for *S. enterica* subsp. *enterica;* 4401 for *E. coli* and *Shigella* spp.; 3449 for the *M. tuberculosis* complex; 1360 for *P. aeruginosa*; and 1100 for *A. baumannii*. For the seven species, G+C content and genome size are summarized in Table 1. Results showed that the G+C content for *M. tuberculosis* and *P. aeruginosa* was >65%, followed by *S. enterica* and *E. coli*, which showed moderate G+C content. The seven species belonged to Firmicutes (*S. pneumoniae* and *S. aureus*), Proteobacteria (*S. enterica, E. coli, P. aeruginosa*, and *A. baumannii*), and Actinobacteria (*M. tuberculosis*) phyla.

**Table 1:** Summary of the genome dataset.

		Genome	G+C
Species	Count	size (MB)	(%)	Taxonomy
*Streptococcus pneumoniae*	6537	2.1	39.6	Firmicutes; Bacilli; Lactobacillales; Streptococcaceae; Streptococcus; *Streptococcus pneumoniae*
*Staphylococcus aureus* subsp. *aureus*	6282	2.9	32.7	Firmicutes; Bacilli; Bacillales; Staphylococcaceae; Staphylococcus; *Staphylococcus aureus*
*Salmonella enterica* subsp. *enterica*	4527	4.8	52.0	Proteobacteria; Gammaproteobacteria; Enterobacteriales; Enterobacteriaceae; Salmonella; *Salmonella enterica*
*Escherichia coli* (*Shigella* spp.)	4401	5.1	50.5	Proteobacteria; Gammaproteobacteria; Enterobacteriales; Enterobacteriaceae; Escherichia; *Escherichia coli*
*Mycobacterium tuberculosis* complex	3449	4.4	65.5	Actinobacteria; Actinobacteria_c; Corynebacteriales; Mycobacteriaceae; Mycobacterium; *Mycobacterium tuberculosis*
*Pseudomonas aeruginosa*	1360	6.7	66.1	Proteobacteria; Gammaproteobacteria; Pseudomonadales; Pseudomonadaceae; Pseudomonas; *Pseudomonas aeruginosa*
*Acinetobacter baumannii*	1100	4.0	39.0	Proteobacteria; Gammaproteobacteria; Pseudomonadales; Moraxellaceae; Acinetobacter; *Acinetobacter baumannii*

### Distribution of ANI Values

For each strain, we calculated ANI compared with that of its corresponding type strain, with these values used to validate species taxonomy based on whole-genome sequences. The resulting ANI values were distributed between 95.9648 and 99.9996% (Supplementary Figure S2A) and within generally recommended ranges for prokaryotic species, which indicated that all genomes agreed with their original taxonomic information (Richter and Rosselló-Móra, 2009; Kim et al., 2014). The highest median ANI was found for the *M. tuberculosis* complex (99.8333%) and *A. baumannii* had the lowest median ANI (97.7098%). Interestingly *E. coli* and *S. aureus* showed relatively large interquartile ranges (IQRs) for their ANI values, followed by the IQRs for *S. enterica* and *P. aeruginosa*. IQRs are used to measure ANI variation, with differences in IQRs indicating substantial differences in genetic diversity across species.

We then evaluated the distribution of ANI values. For each strain, we calculated ANI compared with that of its corresponding type strain and sorted the values in descending order (Supplementary Figure S2B). We found that *E. coli* and *S. aureus* displayed relatively wide ranges of ANIs, which corresponded to both having the largest IQRs (Supplementary Figure S2A). We expected that genomes belonging to the two species would show a large degree of diversity. Interestingly, *S. aureus* showed four points indicating sudden drops in ANI values, suggesting that its strains might be divided into approximately four distinct groups. Both *S. enterica* and *P. aeruginosa* showed ANI values distributed within a relatively moderate range, although the actual distribution showed differences. The ANI values of *S. enterica* were distributed evenly, whereas few *P. aeruginosa* strains were distributed within a close distance to the type strain. Although the *M. tuberculosis* complex comprised genomes from five different species name, the ANI values of the observed strains showed the smallest distance from the type strain, suggesting that their genomes were the most genetically homogeneous.

### Summary of the Pan-Genome and Core Genome

The core genome and the pan-genome of the seven species were constructed according to their clustered orthologous proteins (Table 2). Each species had distinctly different core-genome and pan-genome sizes and compositions, with no clear association found between the number of genomes calculated and the size of the pan-genome. For example, *S. aureus* had the second largest number of genomes (6282); however, it also displayed the smallest number of gene clusters constituting the pan-genome. On the other hand, the *E. coli* and *Shigella* spp. group with only 4401 genomes had the largest pan-genome (128,193 gene clusters). Table 2 also shows the relative proportion of genes belonging to the core genome as compared with the average number of genes (>50% in all seven species). A minimal relative proportion of the core genome was observed for *E. coli* (53%), although it had the largest pan-genome size. The second smallest relative proportion of the core genome was observed for *S. pneumoniae* (55%), but its difference with *E. coli* was very small. These were followed by *A. baumannii*, *S. enterica*, and *P. aeruginosa*. *M. tuberculosis* had an average of 4094 genes, with 81.66% of these belonging to the core genome.

**Table 2:** Summary of the core and pan-genome.

				Pan-	Sum of
	Average no.	Core	Percentage	genome	branch
Species	of genes	size	core (%)	size	length
*Streptococcus pneumoniae*	2076	1143	55.06	71,181	14.46
*Staphylococcus aureus* subsp. *aureus*	2668	2026	75.94	22,133	7.59
*Salmonella enterica* subsp. *enterica*	4606	3281	71.23	55,739	7.59
*Escherichia coli* (*Shigella* spp.)	4889	2608	53.34	128,193	9.73
*Mycobacterium tuberculosis* complex	4094	3343	81.66	33,490	4.12
*Pseudomonas aeruginosa*	6173	4464	72.31	49,744	5.69
*Acinetobacter baumannii*	3838	2355	61.36	29,845	5.08

**Figure 1:**
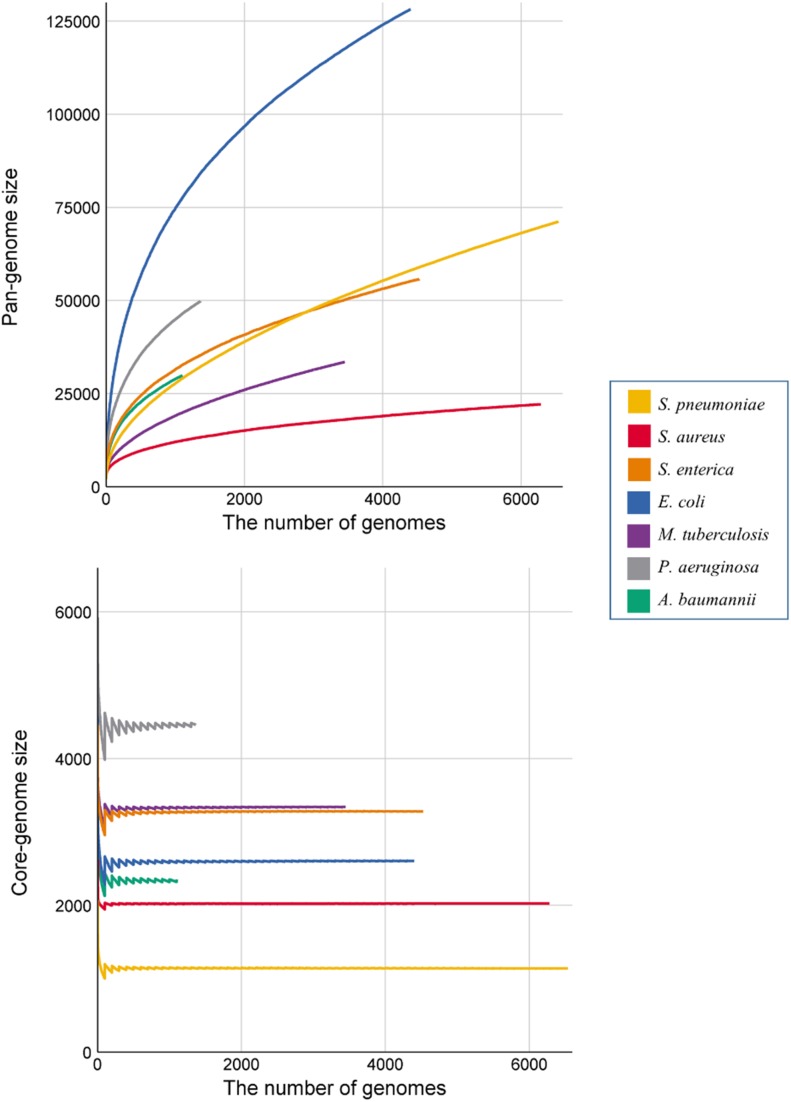
Growth curve of the pan- and core genomes of all seven species. Each color denotes the seven species. Each of the values represent the average number of core- and pan-genome sizes from 100 randomly generated strain orders. In the core-genome graph, the fluctuation at the beginning occurs, because the core-genome cut-off value was fixed at the ratio of the number of genomes used.

Figure 1 shows the pan-genome and core-genome sizes relative to the number of genomes for each species. We observed substantial differences in the growth curves of the pan-genome and core-genome sizes, as well as their slopes, according to the number of genomes among species. The pan-genome size increased in all genomes along with an increase in the number of genomes, and the slopes increased initially before gradually decreasing after 100 genomes. *E. coli* showed the fastest growth rate in size and nearly maintained this rate until the end of the calculation, and three species (*A. baumannii*, *S. enterica*, and *S. pneumoniae*) showed similar increases. Interestingly, the pan-genome size of *S. enterica* was larger than that of *S. pneumoniae*, but only when the number of genomes was <3000. In terms of core-genome size, *P. aeruginosa* showed the largest fluctuation, and *S. aureus* showed the least fluctuation, with all species eventually converging to certain core-genome sizes.

### Openness of the Seven Species

The size of the gene pool for each species and the amount that it increases upon the addition of external genomes can be used to predict whether the species is open or closed. We used a Heaps’ law to measure the pan-genome size (Table 3) using exponent γ values of 0.4964, 0.3292, 0.3731, 0.3750, 0.4350, 0.3491, and 0.3395. When γ > 0, the size of the pan-genome monotonically increased without converging as external genes were added, indicating that the pan-genomes of all the species were open.

**Table 3:** Fitting result of Heaps’ law.

Species	κ	γ	Open/closed
*Streptococcus pneumoniae*	904.1 ± 1.1364	0.4964 ± 2e-04	Open
*Staphylococcus aureus* subsp. *aureus*	1236.0 ± 1.6608	0.3292 ± 2e-04	Open
*Salmonella enterica* subsp. *enterica*	2403.3 ± 2.2146	0.3731 ± 1e-04	Open
*Escherichia coli* (*Shigella* spp.)	5559.0 ± 9.5563	0.3750 ± 2e-04	Open
*Mycobacterium tuberculosis* complex	958.3 ± 3.2618	0.4350 ± 4e-04	Open
*Pseudomonas aeruginosa*	4022.6 ± 5.5498	0.3491 ± 2e-04	Open
*Acinetobacter baumannii*	2772.4 ± 2.5247	0.3395 ± 1e-04	Open

### Comparison of Functional Categories Between Genes in the Core and Accessory Genomes

Clusters of orthologous groups (COGs) for the core and accessory genomes were compared in order to identify functional differences among species (Supplementary Figure S3). With the exception of genes with unknown functions and unassigned categories, the enriched genes in the core genome were involved in metabolism, such as amino acid transport and metabolism (E) and carbohydrate transport and metabolism (G). On the other hand, the enriched genes in the accessory genome were included in information storage and processing, such as replication, recombination, and repair (L), transcription (K), and cell-wall/membrane/envelope biogenesis (M). In *S. pneumoniae*, the defense mechanism (V) category ranked in the top five, and both core and accessory genomes had a high proportion of genes involved in replication, recombination, and repair (L), which was not observed in other species. In most species, amino acid transport and metabolism (E) was the most abundant category in the core genome, whereas in the core genome of *M. tuberculosis*, transcription (K) was the most abundant category. Another unusual feature was that *S. enterica* harbored a significant proportion of genes involved in metabolism-associated categories, even in the accessory genome.

### Comparison of Patterns of Gene Sharing Between the Seven Species

**Figure 2:**
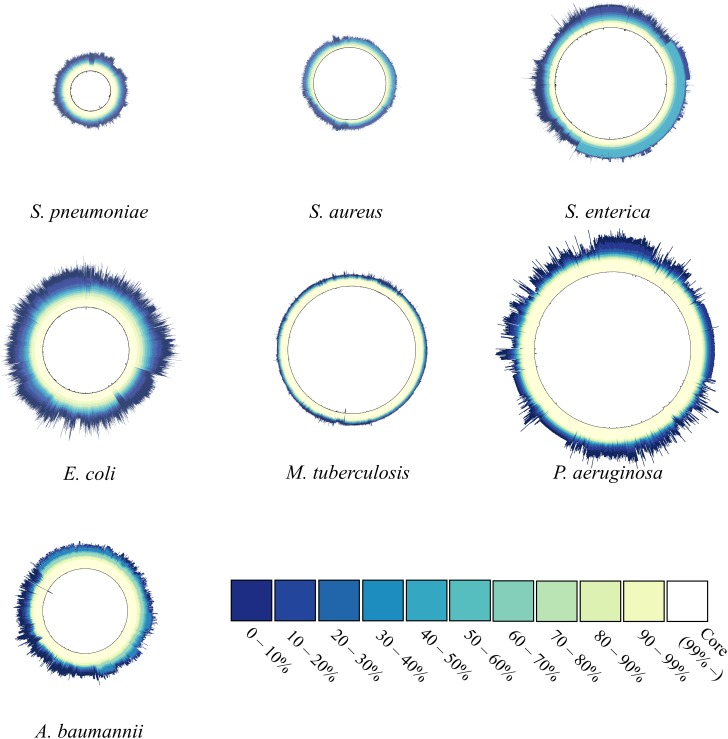
Gene-sharing ratio within species. The number of genes in one strain corresponds to the radius of the circle. For each gene, the ratio of the number of strains carrying the gene in the total strains of one species was calculated and defined as the gene-sharing ratio. The ratio was colored according to 11 different colors depending on the value. The lighter the color (closer to white), the more commonly shared gene is within the strains If certain genes were observed in at least ≥ 99% of strains, the genes were categorized into core genomes (white). The ratio reveals the composition of the gene-pool structure.

Figure 2 shows the proportion of common or uncommon genes among strains. We calculated and compared the number of genes shared among strains in each species and used a single circle to represent each species, with its diameter proportional to the number of genes belonging to the species. According to our results, the surface of the circle for *P. aeruginosa* had multiple spikes, indicating that the number of genes in its strains showed clear variation. Similarly, *E. coli* and *A. baumannii* showed significant variations in the number of genes among strains, but the remaining four species did not show significant protrusion. Specifically, the five species in the *M. tuberculosis* complex resulted in only a few small spikes, indicating that the variation in genome size among strains was not diverse. Additionally, Figure 2 shows the relative proportion of each gene among the strains. Genes were categorized into 11 different groups according to their relative proportions and represented by 11 different colors. The darker blue implies that the genes are shared by fewer strains, whereas a whiter color implies that the genes are shared by more strains. Therefore, the color pattern of the circle intuitively represents the characteristics of the gene pool. The majority of the genes were included in the category denoting 90 to 99% shared genes and core (vanilla and white color), although their proportions differed. In the case of *M. tuberculosis*, the genes mostly belonged to vanilla- or similarly colored sections. Most of the genes of *P. aeruginosa* belonged to the category denoting >90% shared genes, and the proportion of genes that were neither common nor unique (40 to 60% shared) was very small, although some strains of the species showed considerable uniqueness. In the cases of *S. aureus* and *S. enterica*, there was minimal difference in the number of genes depending on the strains, but the differences were divided into two patterns depending on whether the strains included genes belonging to the moderately shared gene category. The proportion of the core category (>99%) for *E. coli* was the smallest among the seven species and showed the widest color spectrum.

### Application of the Proposed Models

**Figure 3:**
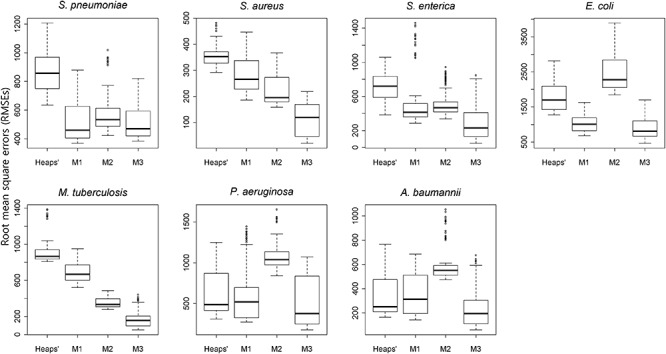
Comparison of the results of each estimation method according to cross-validation. The boxplots illustrate the RMSEs of four different models among the seven species. Using a randomly selected sample order, 10-fold cross-validation was conducted for the Heaps’ law and the proposed model (M1, M2, and M3). The total number of genomes is randomly divided into even 10 subgroups and the estimation model is built with nine of the subgroup data. By measuring the RMSEs between the pan-genome size estimated by the candidate model and the actually observed pan-genome size, the accuracy of each model was evaluated. In all species, the M3 model outperformed the others upon comparison of the median RMSEs.

Heaps’ law and the proposed models (M1, M2, and M3) were applied to estimate the pan-genome property. A 10-fold cross-validation was applied to each species, and box plots for RMSEs for different methods are provided in Figure 3. The results showed that the RMSEs were dependent upon the method, with the worst performance observed from M2 and Heaps’ law, although their performance was affected by species. M3 had the smallest RMSE and achieved the highest accuracy for its prediction of pan-genome size (Figure 3). Additionally, the gap between M2 and Heaps’ law was substantial, as both models use genome size to predict pan-genome size, whereas the M3 model uses an additional covariate (phylogenetic distance). Furthermore, the RMSEs obtained from use of the conventional method were larger, especially for large groups, such as *S. enterica* and *S. aureus*. These results revealed that the phylogenetic distances provided valuable information for predicting pan-genome size.

**Figure 4:**
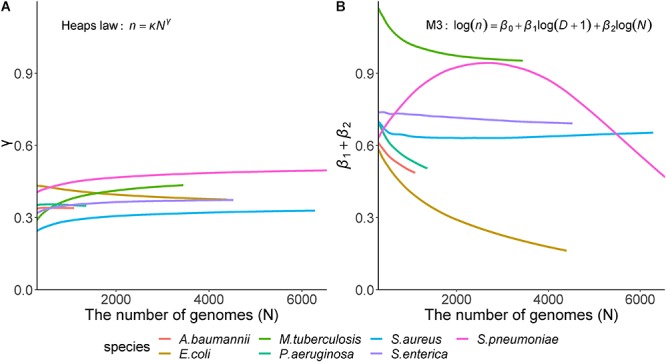
Relationship between genome size and model parameters. Heaps’ law and model M3 that showed the best results according to 10-fold cross-validation were used for comparison. The Y-axis shows the model parameters: **(A)** Heaps’ law parameter (γ); **(B)** Sum of β_1_, which is the coefficient of the tree distance, and β_2_, the genome coefficient in the proposed model (M3). Each color indicates the seven species. In Heaps’ law, regardless of the species, the parameters showed two patterns that simply increase or decrease, on the other hand, in M3 model, the parameters showed different patterns depending on the species.

To measure the robustness of the parameters in the presence of varying genome number, we gradually increased the number of genomes used in the calculations. Averages of each parameter derived from 100 randomly generated datasets indicated that the proposed model showed constant patterns unlike Heaps’ law that showed two patterns depending on species (Figure 4). The γ values (the parameter of Heaps’ law) never decreased below the closed pan-genome threshold of 0, and their curves were classified into two types by species. One type increased along with the inclusion of additional genomes (*S. pneumoniae, S. aureus, S. enterica*, and *M. tuberculosis*), whereas the other type decreased after a sharp initial increase (*E. coli, P. aeruginosa*, and *A. baumannii*). Otherwise, we observed a different pattern in the proposed model, where the coefficient sum continuously decreased in most species, except *S. pneumonia*, and the patterns associated with the parameters agreed closely with previously observed genetic characteristics and gene-sharing ratios. For *E. coli* and *S. enterica*, the two species showed similar γ values according to the Heaps’ law; however, their parameters showed clearly different patterns using the proposed model. Considering their genetic variation and the growth in pan-genome size, the proposed model clearly revealed the genetic differences between the two species, particularly the flexibility for external genes in *E. coli*.

These results suggested that phylogenetic distance provided valuable information for estimating the parameters for pan-genome size, and that the proposed method was effective at considering species as compared with using a Heaps’ law. Moreover, our findings indicated that pan-genome size was estimated with better accuracy and robustness using the novel method.

## Discussion

Improvements in sequencing technology have made available extensive volumes of genome-sequencing data. However, despite the large amount of data, limited genome sequences have been used in estimation functions to predict pan-genome size, resulting in low levels of accuracy. Only four recent studies used a higher number of genomes to predict pan-genome size: 249 genomes from *A. baumannii*, 282 genomes from *E. coli*, 675 genomes from *Salmonella enteritidis*, and 4893 genomes from *S. enterica* (Chan et al., 2015; Salipante et al., 2015; Feasey et al., 2016; Laing et al., 2017). In the present study, we addressed this limitation by using a total of 27,656 genomes from seven species to construct species-specific pan-genomes and show that a larger number of genomes and the use of phylogenetic distance allowed more accurate estimation of pan-genome size. Additionally, we described the genetic characteristics of each species in the context of pan-genome size, ANI values, and gene-sharing ratios.

Our results showed that *S. pneumoniae*, which had the largest number of genomes, displayed high genetic diversity in both common and uncommon regions along with the second lowest core-genome proportion (55%) and the largest sum of branch length in phylogeny, indicating that the common regions were relatively small, and that high degrees of variation exist in this region. However, the distribution of ANI values were small, showing a difference of only up to 4% and with most strains concentrated near 98.5%. Given that the ANI value suggests similarity between sequence fragments having an identity ≥ 70%, and that a branch length in the strict core-genome phylogeny directly reflects base differences, this result showed that the ANI value and the sum of the branch length were not proportional. According to the gene-sharing ratio, in almost all strains, a high proportion of genes in the category denoted by 0 to 10% explains a large size of its pan-genome, even though the average number of genes in the species is as small as 2,000.

*Staphylococcus aureus* exhibits moderate genetic diversity in its common region and low genetic diversity in its uncommon region, indicating that mutations were accumulated in the sequences of the common genome regions while not accepting or maintaining new genes. This species showed the smallest pan-genome size, and on average, 75% of its genes belonged to the core genome. Although the sum of its branch length was smaller than that of *S. pneumoniae*, the range of ANI values appeared wider than that of *S. pneumoniae*. Therefore, it is possible that the new gene was quickly distributed to other strains or that the strains rarely accepted new genes. Considering that the size of the pan-genome was relatively small, and that the ANI value was widely distributed, it is highly likely that new genes were less likely to be accepted, whereas variations accumulated in the common region. This observation was supported by the relatively low proportion of genes that were rarely shared with other strains (∼0–10%).

*Salmonella enterica* presents moderate genetic diversity both in common and uncommon regions. This was supported by the core-genome proportion, the sum of the branch length, and the distribution of ANI values. According to the gene sharing ratio within strains, *S. enterica* were divided into several distinct subgroups: one of subgroups that had a high proportion of genes in the category 40 to 60%, another subgroup that had not only a high proportion of genes in the category 40 to 60% but also had extra uncommon genes, and still another subgroup that had a low proportion of moderately shared genes. This result supported a previous finding that *S. enterica* species are divided into several subspecies and >2500 serovars (Fierer and Guiney, 2001).

As expected, *E. coli* showed high genetic diversity in both common and uncommon regions. The proportion of the core genome was the lowest (53%) as compared with the average gene count, and the 128,193 genes in the pan-genome were the largest among the seven species. The ANI values of the species were also distributed across the widest range (95.96–99.99%), but the sum of the branch lengths was not large relative to the diversity of its gene pool. For the gene-sharing ratio, the variation in the number of genes within strain was large, with many genes included in a category showing minimal sharing with other strains, which clearly supported the high genetic diversity of the species. These results were consistent with a previous finding that *E. coli* widely accepted external genes in order to adapt to ecological niches and also includes a population that exhibits a significant variation in ecological niche (Chen et al., 2006). During the adaption process, some commonly existing genes were considered no longer important according to altered environmental conditions, eventually leading to loss.

The genetic diversity of *M. tuberculosis* was the lowest among the seven species, with the results indicating that this species maintained its genetic disposition with minimal changes. This was clearly represented in the gene-sharing ratio, which showed a high number of shared genes and few unique genes. Similarly, most of the ANI values were concentrated close to those of the type strain, and the cumulative sum of the branch lengths was the shortest. These results indicated that most of the genes were shared with other strains, which might be related to characteristics involved in forming the *M. tuberculosis* complex.

*Pseudomonas aeruginosa* showed moderate genetic diversity in both common and uncommon regions. Despite the relatively large genome size of the species and its inclusion of >6000 genes, the absolute number of genes that constituted the core genome was as high as 71%. The sum of the branch length and the distribution of ANI values supported the moderate variation in its genetic content. This result was similar to that of other gene-acquiring species, such as *S. enterica*; however, we observed unique characteristics in the gene-sharing ratio pattern. Interestingly, there was a large deviation in the acquisition of genes from each strain, with this feature resulting in a relatively high number of unique genes and a relative low number of shared genes. This suggested that the species accepted the different degrees of external genes, and it is highly likely to utilize these genes as strain-specific pathways.

*Acinetobacter baumannii* showed high genetic diversity in common regions and moderate genetic diversity in uncommon regions. The proportion of genes in the core genome (61%) was close to that observed in species showing high genetic diversity (*E. coli* and *S. pneumonia*); however, the sum of the branch length was similar to or larger than that in other species, although this species included the smallest genome. The ANI values were mainly distributed within the 97 to 98% range, which was relatively wide given the number of genomes. These results suggested that most of the *A. baumannii* strains harbored considerably different genetic contents relative to their type strain. The gene-sharing ratio displayed a pattern roughly divided into two groups, with one containing fewer genes that were less frequently shared with other strains and the other containing more genes from a non-core genome that was shared with other strains. This might suggest that the species consists of two genetically distinct groups: one flexible enough to accept new genes in external environments and another that exhibits a robust genetic makeup.

Our results demonstrated the presence of distinct variations in genomic composition between species, although the distribution of ANI values confirmed that all analyzed genomes in this study comprised a single species and were included in a one-species boundary. Additionally, the proportion of the core genes relative to the total genes in the strains was similar to that reported in previous studies. This indicates that the number of core genomes has remained constant, even though there were slight differences in the algorithm used to identify orthologs and the number of genomes used.

We applied a Heaps’ law to determine whether the pan-genomes of the seven species were open or closed, with all alpha estimates less than one. These results indicated that the gene pool of all species is open. Some of these results agree with those of previous studies. Salipante et al. (2015) reported the openness of *E. coli* using 283 extra intestinal pathogenic strains. Additionally, Donati et al. (2010) and Paul et al. (2016) showed that *S. pneumoniae* and some sub-group of *S. enterica* displayed ever-growing sizes of pan-genomes. However, results for two other species (*A. baumannii* and *S. aureus*) have been inconsistent. Liu et al. (2014) showed that *A. baumannii* has an open pan-genome, whereas Chan et al. (2015) showed that a pan-genome of the species appeared to be closed in its 249 genomes after 1 year. Similarly, Tettelin et al. (2008) reported that *S. aureus* had a closed pan-genome, although Bosi et al. (2016) reported that the species had an open pan-genome. There are multiple potential reasons for such inconsistencies. First, the number of genomes used in previous studies was <200, with this number frequently differing between studies. Given that pan-genome size is heavily influenced by the properties of the genomes used, this variation would likely result in inconsistencies. Second, the pan-genomes were not created using the same algorithm across all studies. Finally, by using a large number of different strains, relatively new genes were often included, which resulted in open pan-genomes. In the present study, our results showed that the openness of some species was incompatible with earlier findings for many of the same reasons. Moreover, based on pan-genome size, the Heaps’ law suggested by Tettelin et al. (2008) and utilized as a reference standard for openness or closeness could not provide a clear estimation. A possible explanation for this is because even when using the same number of strains from the same species, each member harbors different genetic variability, which is not uniform. For example, suppose that there are two independently evolved subgroups within a species. The difference between creating a pan-genome using only strains from within the same subgroup and those without is obvious. Additionally, as the sequences of more genomes become available, the probability of finding strains with unique genetic properties relative to those in existing species increases according to the accumulated lateral gene transfer. To overcome such limitations, we suggested a novel model that reflects evolutionary time between species and considers the cumulative sum of the branch length. By applying this model, we accurately estimated the parameters of pan-genome size using large amounts of genome data. Moreover, we confirmed that the model was less affected by genome size in most species after the addition of a certain number of genomes. Assuming that *E. coli* and *P. aeruginosa* converge to a single value when a higher number of genomes are used, it is likely that we will observe more certainty associated with *S. pneumoniae* characteristics if additional sampling is performed.

It is difficult to measure the gene pool of a species with a snap shot called genome sequencing because bacteria continue to add or drop genes. In addition, sequencing errors and draft genomes make accurate estimation more difficult, and sequencing bias due to clonal breaks can affect the size of the estimated gene pool. Nonetheless, based on our findings, we have extended the understanding of species using the proposed method, and in particular, this has minimized the sampling bias associated with genome sequencing due to preference for pathogenic strains. However, our findings need to be verified with additional genomes and other species.

## Author Contributions

S-CP, KL, and JC conceived the project and designed the experiments. S-CP and YK implemented the main algorithm. S-CP wrote the manuscript. S-CP, KL, SW, and JC reviewed the manuscript.

## Conflict of Interest Statement

The authors declare that the research was conducted in the absence of any commercial or financial relationships that could be construed as a potential conflict of interest.

## Footnotes

1 https://www.ncbi.nlm.nih.gov/genbank/

2 http://lowelab.ucsc.edu/tRNAscan-SE/

3 http://hmmer.org/

4 http://eggnogdb.embl.de/

5 https://drive5.com/usearch/manual/uclust_algo.html

6 https://mafft.cbrc.jp/alignment/software/

7 http://www.microbesonline.org/fasttree/
