# Supplementary material for: Large-Scale Genomics Reveals the Genetic Characteristics of Seven Species and Importance of Phylogenetic Distance for Estimating Pan-Genome Size
Source: Front Microbiol. 2019 Apr 24;10:834. doi: 10.3389/fmicb.2019.00834 (PMC6491781; doi:10.3389/fmicb.2019.00834)
Supplement: Supplementary file 2 [file Data_Sheet_1.PDF]

Supplementary Material

Supplementary Material

1 Supplementary Figures and Tables

1.1 Supplementary Figures

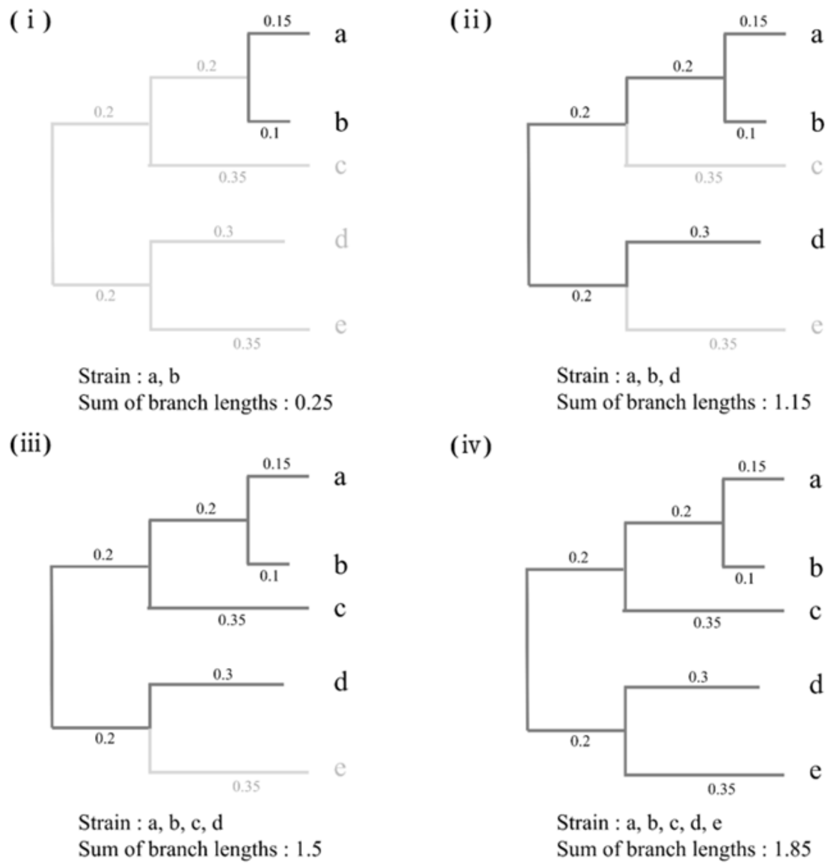

Supplementary Figure S1. Cumulative sum of the branch lengths. When a single strain is added, all branch lengths from the root to the corresponding node are added. This procedure was repeated until all of the nodes were included.

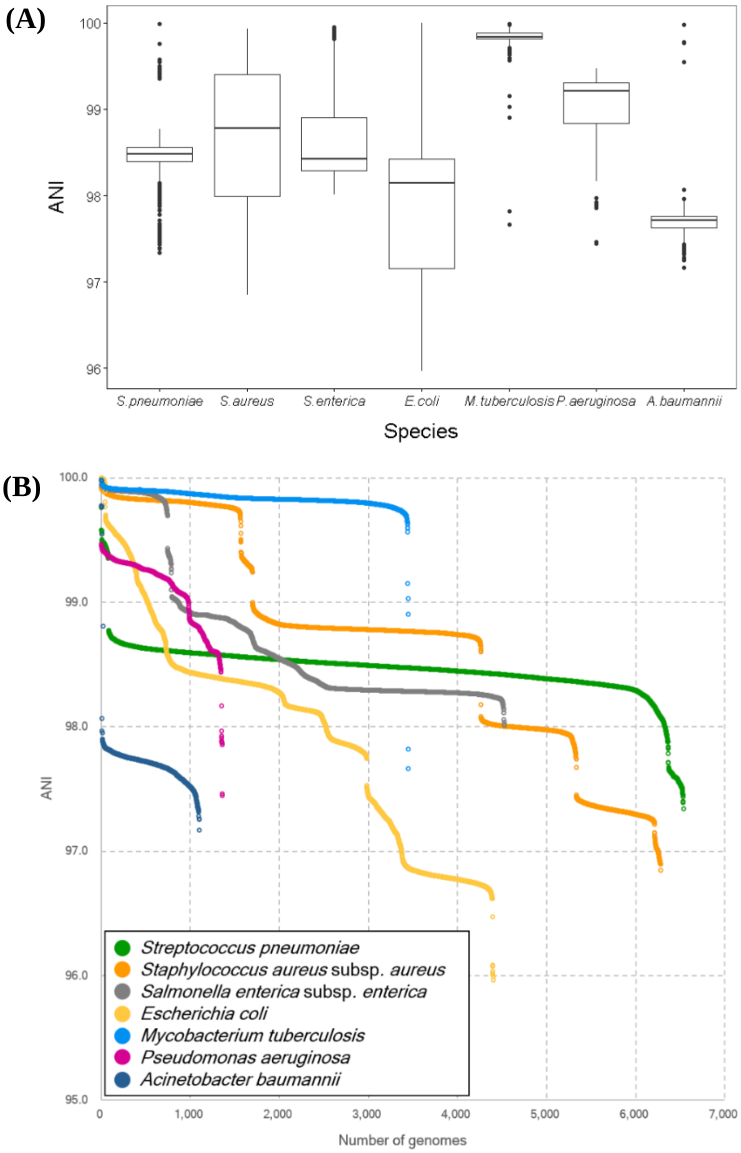

Supplementary Figure S2. Distribution of ANI values in the seven species. (A) Box plot of ANI values for each type strain in the seven species. If the genetic diversity of the species differs but all ANI values are distributed within 95%, a boundary for the species delineation. (B) The descending order of ANI values shows the genetic diversity of the seven species. *M. tuberculosis* strains were distributed continuously, except for a few strains; however, *S. aureus* strains were divided into several discrete groups.

	S.pneumoniae		*S.aureus*		*S.enterica*		*E.coli*		*M.tuberculosis*		*P.aeruginosa*		*A.baumannii*	
RANK	Accessory	Core	Accessory	Core	Accessory	Core	Accessory	Core	Accessory	Core	Accessory	Core	Accessory	Core
1	[L] Replication, recombination and repair	[J] Translation, ribosomal structure and biogenesis	[L] Replication, recombination and repair	[E] Amino acid transport and metabolism	[F] Nucleotide transport and metabolism	[E] Amino acid transport and metabolism	[L] Replication, recombination and repair	[E] Amino acid transport and metabolism	[G] Carbohydrate transport and metabolism	[K] Transcription	[L] Replication, recombination and repair	[E] Amino acid transport and metabolism	[L] Replication, recombination and repair	[E] Amino acid transport and metabolism
2	[G] Carbohydrate transport and metabolism	[E] Amino acid transport and metabolism	[M] Cell wall/membrane/ envelope biogenesis	[J] Translation, ribosomal structure and biogenesis	[L] Replication, recombination and repair	[G] Carbohydrate transport and metabolism	[M] Cell wall/membrane/ envelope biogenesis	[G] Carbohydrate transport and metabolism	[L] Replication, recombination and repair	[I] Lipid transport and metabolism	[K] Transcription	[K] Transcription	[K] Transcription	[C] Energy production and conversion
3	[V] Defense mechanisms	[G] Carbohydrate transport and metabolism	[K] Transcription	[P] Inorganic ion transport and metabolism	[K] Transcription	[K] Transcription	[K] Transcription	[C] Energy production and conversion	[N] Cell motility	[E] Amino acid transport and metabolism	[U] Intracellular trafficking, secretion, and vesicular transport	[C] Energy production and conversion	[M] Cell wall/membrane/ envelope biogenesis	[K] Transcription
4	[M] Cell wall/membrane/ envelope biogenesis	[L] Replication, recombination and repair	[E] Amino acid transport and metabolism	[G] Carbohydrate transport and metabolism	[M] Cell wall/membrane/ envelope biogenesis	[C] Energy production and conversion	[G] Carbohydrate transport and metabolism	[P] Inorganic ion transport and metabolism	[K] Transcription	[C] Energy production and conversion	[M] Cell wall/membrane/ envelope biogenesis	[P] Inorganic ion transport and metabolism	[P] Inorganic ion transport and metabolism	[J] Translation, ribosomal structure and biogenesis
5	[E] Amino acid transport and metabolism	[P] Inorganic ion transport and metabolism	[P] Inorganic ion transport and metabolism	[K] Transcription	[G] Carbohydrate transport and metabolism	[P] Inorganic ion transport and metabolism	[U] Intracellular trafficking, secretion, and vesicular transport	[K] Transcription	[Q] Secondary metabolites biosynthesis, transport, and catabolism	[L] Replication, recombination and repair	[P] Inorganic ion transport and metabolism	[T] Signal transduction mechanisms	[E] Amino acid transport and metabolism	[P] Inorganic ion transport and metabolism

CELLULAR PROCESSES AND SIGANLING   INFORMATION STORAGE AND PROCESSING   METABOLISM

Supplementary Figure S3. The top five COG categories in the core and accessary genomes. Each color describes the three general functional categories: cellular processes and signaling, information storage and processing, and metabolism. Each box describes the categories in detail.

1.2 Supplementary Tables

Supplementary Table S1. List of genome sequences of *Streptococcus pneumoniae* used in this study.[a]

[a] Genome size, assembly identifier, NCBI designation, and ANI values for the representative strain are summarized.

Supplementary Table S2. List of genome sequences of *Staphylococcus aureus* subsp. *aureus* used in this study.[a]

[a] Genome size, assembly identifier, NCBI designation, and ANI values for the representative strain are summarized.

Supplementary Table S3. List of genome sequences of *Salmonella enterica* subsp. *enterica* used in this study.[a]

[a] Genome size, assembly identifier, NCBI designation, and ANI values for the representative strain are summarized.

Supplementary Table S4. List of genome sequences of *Escherichia coli* and *Shigella* spp. used in this study.[a]

[a] Genome size, assembly identifier, NCBI designation, and ANI values for the representative strain are summarized.

Supplementary Table S5. List of genome sequences of the *Mycobacterium tuberculosis* complex used in this study.[a]

[a] Genome size, assembly identifier, NCBI designation, and ANI values for the representative strain are summarized.

Supplementary Table S6. List of genome sequences of *Pseudomonas aeruginosa* used in this study.[a]

[a] Genome size, assembly identifier, NCBI designation, and ANI values for the representative strain are summarized.

Supplementary Table S7. List of genome sequences of *Acinetobacter baumannii* used in this study.[a]

[a] Genome size, assembly identifier, NCBI designation, and ANI values for the representative strain are summarized.
